# Spiritual needs of adolescents with cancer: a systematic review and meta-synthesis

**DOI:** 10.1186/s12904-025-01904-1

**Published:** 2025-10-15

**Authors:** Jin Ge Wei, Xiao Nan Zhang, Zhi Hong Ni

**Affiliations:** 1 Department of Nursing, Children’s Hospital of Soochow University, No. 92 Zhong Nan Street, Soochow, Jiangsu Province China; 2 School of Nursing, Medical College of Soochow University, No. 1 Shi Zhi Road, Soochow, Jiangsu Province China

**Keywords:** Adolescents, Cancer, Children, Meta-synthesis, Qualitative research, Spiritual needs, Spirituality, Religion

## Abstract

**Background:**

Spirituality is an important component of palliative care, yet research focusing on adolescents remains in its early stages, with few studies addressing the spiritual needs of this group. The study aimed to integrate the spiritual needs of adolescents with cancer and provide a reference for formulating personalised spiritual care plans for this demographic.

**Methods:**

Eleven databases (Wanfang Data, China National Knowledge Infrastructure, VIP Information Resources System, China Biology Medicine, Web of Science, MEDLINE, EMBASE, Cochrane Library, CINAHL, PubMed, and PsycINFO) were searched for relevant qualitative studies published from the inception of the database to March 2025. We utilized the Australian JBI Quality Assessment Criteria for Qualitative Research to evaluate the quality of the included studies and utilized thematic analysis approach for data analysis. Presentation of this synthesis adhered to the PRISMA guideline.

**Results:**

Eight papers were included, yielding 13 sub-themes. Five integrative themes were summarised, including ‘Being autonomous’, ‘Being connected’, ‘Finding the meaning of life’, ‘Having a positive attitude’, and ‘Dealing with death’.

**Conclusions:**

Spirituality is an important part of the lives of adolescents with cancer, significantly affecting their spiritual health and quality of life. However, the spiritual needs of this group are often overlooked. Paediatric healthcare workers should facilitate access to spiritual care and support them through comprehensive and personalised interventions.

**Supplementary Information:**

The online version contains supplementary material available at 10.1186/s12904-025-01904-1.

## Background

Cancer is the leading cause of disease-related death among adolescents [1]. According to the World Health Organisation, approximately 400,000 adolescents aged 0–19 years are diagnosed with cancer globally each year [2]. Although innovations in medical technology and treatment have improved survival rates for adolescents with cancer [3], they continue to experience physical impairments and psychological challenges associated with long-term treatment [4]. Some adolescents may develop spiritual needs in response to the stress of illness; however, the expression of these needs is shaped by individual differences and cultural contexts [5, 6].

Spirituality is often described in the literature as encompassing three principal components: connectedness, transcendence, and meaning in life [7], though these specific concepts have not been consistently defined. Previous research has posited that spirituality is an intrinsic aspect of human nature, through which individuals explore self-worth, meaning, and purpose in life [8–10]. In contrast to adults, the spirituality of adolescents tends to be more focused on relational awareness, prioritising connections to the self, followed by connections to others, nature, and ultimately to the sacred or transcendent. Furthermore, fostering a connection to self in adolescents, including the cultivation of a sense of meaning, purpose, and joy, is essential [11]. Spirituality has often been closely associated with religion. Lazenby [12], drawing on the philosophies of Frege and James, suggested that “spirituality” and “religion” actually refer to the same core concept, e.g. the individual’s immediate response to and experience of life, including the perception of the sacred, the search for ultimate meaning, or connection to the transcendence [13].

Spiritual needs refer to an individual’s deep need for meaning, purpose, inner peace, belonging, and hope in life [14, 15]. Moore et al. [16] found that adolescents with cancer exhibit stronger spiritual needs and significantly outscore their healthy peers on certain spiritual dimensions. Addressing these spiritual needs is closely linked with positive outcomes in adolescents with cancer [15, 17], aiding them in their exploration of meaning and purpose throughout their illness, achieving inner peace, and increasing their sense of hope, which enhances their overall coping mechanisms [17–19].

Religion and spirituality are central to the philosophy of palliative care, and spiritual care is recognised as an important component of both holistic and palliative care [20]. In recent years, spirituality has also gained increasing attention in paediatric hospice and critical care, with a growing body of literature exploring the spiritual needs and spiritual care of adolescents with cancer. However, existing qualitative research has yet to integrate the dispersed evidence through meta-synthesis methods. Bakker et al. [21] synthesised the spiritual needs of children with chronic illness, stating that their spirituality is shaped by a search for identity, and that they require strong interpersonal relationships, a supportive medical environment, and positive spiritual coping. However, this meta-synthesis did not differentiate between specific illnesses, and the inclusion of predominantly US-based studies with most participants self-identifying as religious may have resulted in an understanding of spirituality that leans too heavily on religious frameworks and overlooks non-religious expressions of spirituality.

We explored the characteristics and expressions of the spiritual needs of adolescents with cancer from different cultural backgrounds. By comparing various religious traditions found in different cultures (such as Christianity and Islam), as well as non-religious spiritual expressions, we gained insights into the influence of culture and belief on the spiritual needs of adolescent cancer patients.

## Aims

This study aimed to synthesis qualitative evidence on the spiritual needs of adolescents with cancer, with attention to potential variations across cultural or religious contexts. Our findings may serve as a reference for clinicians and nurses in developing targeted interventions to support adolescents with cancer and enhance their coping processes.

## Methods

### Design

Meta-synthesis is a systematic research method that compares, transforms, and analyses original findings to generate new interpretations of a research question, thereby deepening understanding [22]. This study was conducted under the guidance of Purssell et al. [23], following the preferred reporting items of the system evaluation and meta-synthesis (PRISMA) checklist guidelines [24]. The included literature was independently appraised using the JBI Quality Assessment Criteria for Qualitative Research [25] and used thematic analysis [26] to code and group the results of the qualitative research.

### Search methods

The databases Wanfang Data, China National Knowledge Infrastructure, VIP Information Resources System, China Biology Medicine, Web of Science, MEDLINE, EMBASE, Cochrane Library, CINAHL, PubMed, and PsycINFO were utilised. The search period spanned from the inception of each database to March 2025. According to the PICo framework, two elements—adolescents with cancer and spiritual needs—were used. The research was categorised as “qualitative research”. Search terms were employed with the Boolean operators ‘AND’ and ‘OR’ to facilitate multiple combination searches. The strategies used in this study are listed in Table S1 in the Supplementary Material.

We define adolescence primarily as the developmental transition period between childhood and adulthood, using the WHO’s chronological criterion (10–19 years) [27]. However, given that cancer patients’ developmental trajectories are frequently altered by disease or treatment (e.g. precocious puberty or delayed development), we extended the age range to 5–21 years. This adjustment reflects the continuity of spiritual needs in clinical practice. The inclusion criteria were as follows: (a) Research participants included healthcare professionals, adolescents with cancer, and caregivers; (b) The phenomenon under study was the spiritual needs of adolescents with cancer; (c) The research scenario involved exploring the perspectives of adolescents with cancer on their spiritual needs from their own viewpoints; (d) The primary research methodology employed qualitative approaches, including phenomenological research, grounded theory research, and exploratory research. (e) Patients aged 5–21 years with a cancer diagnosis; (f) Minimum JBI quality: ≥6 ‘Yes’ responses (Class B or A). The exclusion criteria were as follows: (a) inability to access the full text; (b) articles not written in English or Chinese; (c) Studies scoring ≤ 5 ‘Yes’ responses (Class C).

### Study selection

EndNote 21 was utilised to import articles and identify duplicates. Initially, the first author screened titles and abstracts based on inclusion and exclusion criteria. Records meeting the inclusion criteria were then accessed in full text. Subsequently, these full texts were jointly evaluated by two researchers (WJG and ZXN) to determine the final selection of eligible articles.

### Quality appraisal

The Australian JBI Quality Assessment Criteria for Qualitative Research [25] was used to assess the quality of the included literature, which comprised 10 assessment indicators. The outcomes were ‘yes’, ‘no’, ‘unclear’, and ‘not applicable’. Studies were graded as: Grade A (≥ 8 ‘Yes’ responses, low bias risk), B (6–7 ‘yes’ responses, moderate risk), or C (≤ 5 ‘yes’ responses, high risk). Two researchers (WJG and ZXN) independently reviewed each study in accordance with these criteria. Studies in which at least six questions were answered with ‘yes’ were considered for inclusion in this meta-synthesis. In cases of disagreement between the two researchers’ evaluations, a third researcher (NZH) was consulted to achieve a consensus. The results of the quality evaluation are presented in Table 1.

**Table 1:** Methodological quality appraisal of included studies (*n* = 8)

Article codes	Author (year)	Q1	Q2	Q3	Q4	Q5	Q6	Q7	Q8	Q9	Q10	Grade
(A1)	Proserpio et al. [28]	U	Y	Y	Y	Y	N	U	Y	Y	Y	B
(A2)	Liu et al. [29]	Y	Y	Y	Y	Y	N	Y	Y	Y	Y	A
(A3)	Juskauskiene et al. [30]	Y	Y	Y	Y	Y	Y	Y	Y	Y	Y	A
(A4)	Alvarenga et al. [31]	U	Y	Y	Y	Y	N	N	Y	Y	Y	B
(A5)	Zeighamy and Sadeghi [32]	Y	Y	Y	Y	Y	N	N	Y	Y	Y	A
(A6)	Mahayati [33]	Y	Y	Y	Y	Y	N	N	Y	Y	Y	A
(A7)	Alshakhshir et al. [34]	Y	Y	Y	Y	Y	Y	Y	Y	Y	Y	A
(A8)	Kamper et al. [35]	Y	Y	Y	Y	Y	N	U	Y	Y	Y	A

JBI’s Critical Assessment Checklist for Qualitative Research

Q1: Are Philosophical Foundations and Methodology Consistent?

Q2: Is the research objective or question consistent with the method used?

Q3: Is the data collection method consistent with the method?

Q4: Is the representation and analysis of data consistent with the method?

Q5: Is the explanation consistent with the method used?

Q6: Is there a statement regarding the cultural or theoretical positioning of researchers?

Q7: Did the researchers influence the research, and vice versa?

Q8: Are participants and their voices sufficiently representative?

Q9: According to current standards, is the research ethical, or, in the case of recent research, is there evidence to suggest that ethical approval from appropriate institutions?

Q10: Do the conclusions drawn from the research report come from the analysis or interpretation of the data?

Evaluation result: “Y”: Yes; “N”: No; “U”: unclear; “N/A”: Not applicable

### Data extraction and synthesis

Information extracted included the year of publication, author, country, participants’ age, research method, scenario, phenomenon of interest, and research results. We employed thematic analysis [26] guided by the principle of Smith’s method of interpretive phenomenological analysis [36], with two researchers (WJG and ZXN) systematically synthesising the data using a three-stage method [37]: In the first stage, each reviewer independently examined all textual extracts from the eight included studies (coded sequentially as A1 to A8) to identify statements pertaining to spiritual needs of adolescents with cancer, conducting line-by-line coding labelled with the corresponding study codes (e.g. A1, A2, etc.). The original texts were revisited iteratively until theoretical saturation was achieved (e.g. no new codes emerged). In the second stage, the researchers grouped the initial codes based on their semantic similarities to generate preliminary themes. In cases of disagreement, a consensus was reached through discussion and re-examination of the source material. In the third stage, the derived themes were cross-checked against the original data to ensure conceptual consistency, culminating in the final thematic framework.

### Ethical consideration

As this study was a meta-synthesis, ethical approval was not required.

## Results

### Search outcome

**Fig. 1:**
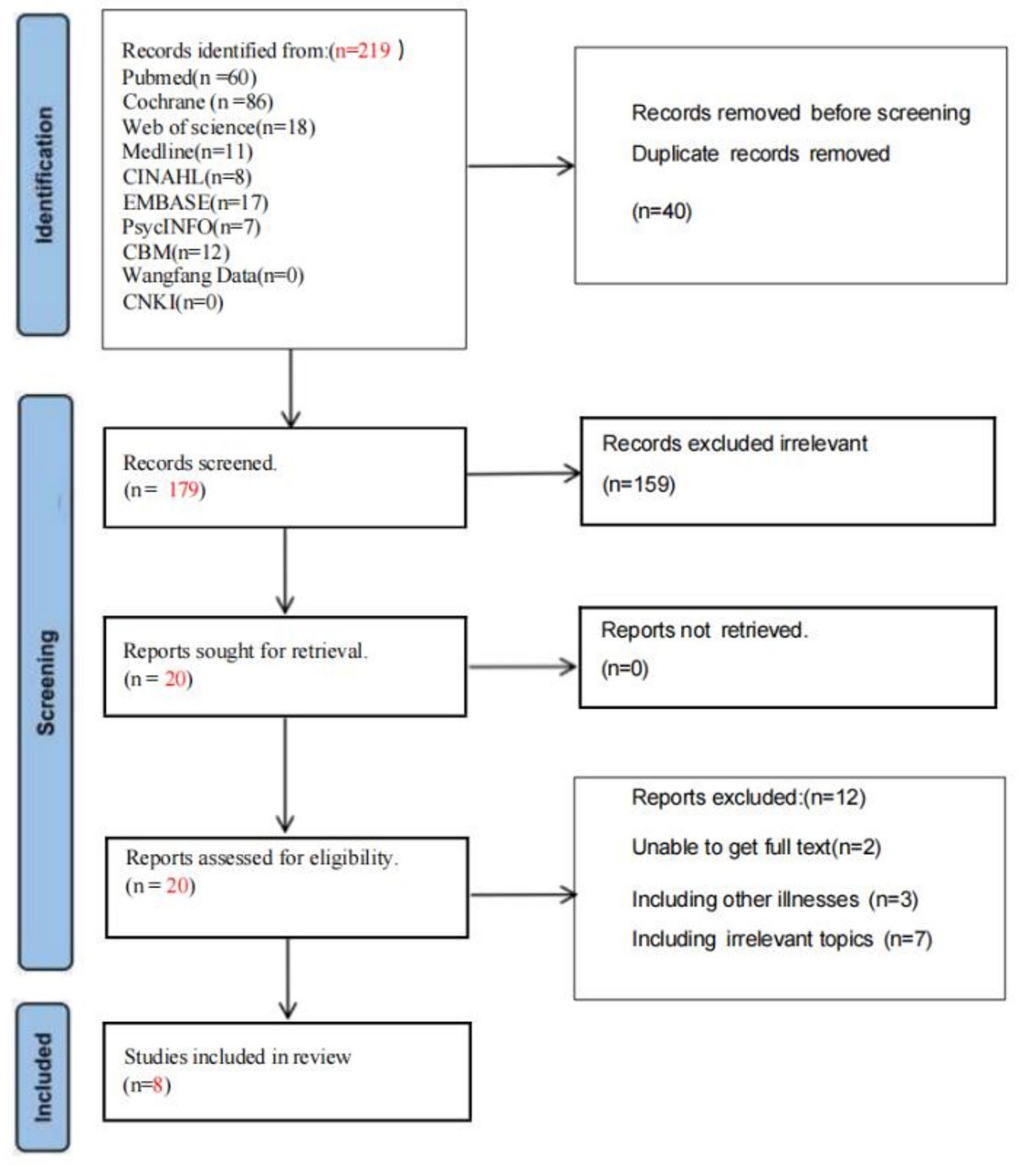
Flowchart of search results and study selection

A total of 219 articles were initially retrieved from the preliminary search. Using EndNote 21, 40 duplicate studies were identified and excluded, resulting in 179 articles eligible for the meta-synthesis. Of these, 159 were subsequently excluded based on the specified criteria. The remaining 20 studies were thoroughly reviewed, with 12 further excluded. Ultimately, eight articles published between 2010 and 2025 were selected for inclusion, and were incorporated into the quality appraisal. The process of literature screening is depicted in Fig. 1.

### Quality appraisal of the included studies

The eight studies included reported an affirmative “yes” on seven or more questions, indicating good quality shown in Table 1. Consequently, all eight studies were incorporated into the final qualitative analysis.

### Study characteristics

Table 2 summarises the basic characteristics of the included literature. The eight studies encompassed 217 participants, including adolescents with cancer (*n* = 210), their families (*n* = 1), and healthcare workers (*n* = 6). However, the studies did not focus on comments from caregivers and healthcare professionals. The participants included adolescents with cancer aged between 5 and 21 years old. The countries involved in the study were Italy [28], China [29], Lithuania [30], Brazil [31] Iran [32], Indonesia [33], and the United States [34, 35]. All studies aimed to explore the spiritual needs of adolescents with cancer.

**Table 2:** Summary table of data extraction

Author (year) country and article codes	Participants’ age	Research method/Sampling method	Scene	Phenomena of interest	Key findings
Proserpio et al. (2020) [28] Italy(A1)	15–24 years	Semi-structured interview/Purposeful sample	Hospital	Spiritual Needs of Adolescents with cancer	1.The need for a connection with others2.The need to find meaning3.The need to sustain hope4.The need to deal with dying and death
Liu et al. (2022) [29] China (A2)	8–17 years	Semi-structured interview/Purposeful sample	Hospital	Spiritual Needs of Chinese Children Hospitalized with Cancer	1.The need for self-exploration2.inner needs3.The need for a connection with others4.The need for a connection with gods, supernatural powers, and fictional characters
Juskauskiene et al. (2023) [30] Lithuania (A3)	5–12 years	Semi-structured interview/Purposeful sample	Hospital	The experience and perception of spiritual lives of children with cancer	1.The need to integrate meaning and purpose in life2.The need to sustain hope3.The need for expression of faithand to follow religious practices4.The need for comfort at the end of life5.The need to connect with family and friends
Alvarenga et al. (2021) [31] Brazilian (A4)	7–18 years	Semi-structured interview/Purposeful sample	Hospital	The spiritual needs of children and adolescents with chronic illnesses and how these needs are met by health professionals during hospitalization.	1.The need to integrate meaning and purpose in life2.The need to sustain hope3.The need for expression of faith and to follow religious practices4.The need for comfort at the end of life5.The need to connect with family and friends.
Zeighamy and Sadeghi (2016) [32] Iran (A5)	15–20 years	Semi-structured interview/Purposeful sample	Hospital	The spiritual/religious needs of adolescents with cancer in Iran	1. The need for a Relationship with transcendence2. The need for a Relationship with the Self3.The need for a relationship with others4.The need for a relationship with the environment and nature
Mahayati (2018) [33]Indonesia (A6)	14–18 years	Semi-structured interview/Purposeful sample	Unknown	Spiritual experiences in adolescents with cancer	1.Accepting their illness2.Believing their illness in God’s will3.Improving spiritual practices4.Expressing empathy to parents5.Maintaining relationships with significant others6.achieving self-actualization
Alshakhshir et al. (2025) [34] USA (A7)	13–19 years	Semi-structured interview/Purposeful sample	Hospital	Describe the essential structure of the process of awakening the spiritual identity as experienced and perceived by adolescents with cancer and explorefactors that facilitate or hinder the process	1.Spiritual Identity2.Spiritual-Religious Foundations3.Connectedness with the Transcendent4.Connectedness with Others
Kamper et al. (2010) [35]USA (A8)	6–17 years	Semi-structured interview/Purposeful sample	Unknown	The responses of children with advanced cancer to a spiritual quality of life (SQL) interview.	1.The need for a connection with others2.The need for a connection with gods, supernatural power3.The need to sustain hope4.The need for returning to a normal life and family environment

Data from Proserpio et al. (2020) (N=33, age 15–24). The 6 cases cited in analysis were aged 16–19, all within the study’s inclusion criteria (5–21 years)

### Themes and subthemes

Thirty-six research themes were obtained from the included studies, and through meta-synthesis, thirteen subthemes and five comprehensive themes were identified: (1) Being autonomous; (2) Being connected; (3) Finding the meaning of life; (4) Having a positive attitude; (5) Dealing with death. (Table 3)

**Table 3:** Coding and grouping table

Synthesized themes	Sub-themes	Research findings
1.Being autonomous	The need to be treated as equals	did not want to be teased about their changed appearance (A2). often not able to join in games (A3). felt like outsiders (A3). give them lots of attention (A8).
	The need to continue life as usual	Having presents, toys and activities helped to ease the emotional burden of being sick and hospitalized (A3). being different from other children increases their unhappiness and isolation (A3). constraints and/or effects of illness make them feel bad (A8).
	The need to be informed about their medical condition	want to hear something real (A2). the interaction with the health professionals contributed to the integration of meaning (A4).
	The need to be in control	the need for a proper unit environment and the need for a proper hospital environment (A5). want to be involved in choosing food preferences (A5).
2.Being connected	The need to maintain relationships	can’t play with friends (A2). pets provide children with unconditional love and caring (A3). family’s presence has a positive effect on hospitalized adolescents (A4, A8). need for a relationship with the family and the need for a relationship with friends and the medical team (A3, A5, A6).
	The need to love and be loved	need to make sure others were happy around them, but at the same time, needing the affirmation of love from friends and family (A2, A3). The support of family and friends is important (A6, A8). All the positive messages from friends and family really helped (A7).
	The need to communicate with others	needed to talk, to try and understand (A1). talking to others makes them feel better and brings strength (A1, A2, A3, A4). prefer to communicate with family over peers (A4)
	The need to connect with transcendence	seeking protection from gods and supernatural powers (A1, A2, A7, A8). learning from Fictional Characters (A2). how connections with God could really be made and could help them (A3). prayers revolved around their need to be healed and to feel well again (A3). need for expression of faith and to follow religious practices (A4). a belief in the existence of God and in the miracle of healing (A4). needed a place to do religious practices in the cancer unit or hospital, to be alone with God and relax by praying (A5) Improving spiritual practice (A6). ponder the afterlife (A8).
3.Finding the meaning of life	The need to accept cancer and seek its meaning	asking existential questions (A1, A2, A4, A6). perceptions regarding their experiences of cancer (A2, A6). the reason of illness (A2). integrated the meaning of the disease and purpose in life with their religious beliefs (A4, A6).
	The need to have a meaningful life	find meaning in life (A2). Self-actualization helps to develop and build self-potency and produce something positive (A6).
4.Having a positive attitude	The need for peace	physically and psychologically tortured the disease and persistent negative thoughts (A2). need for peace(A2). like quiet surrounding (A5).
	The need to sustain hope	helping them to overcome the pain and suffering that resulted from cancer (A2). Optimism helps them focus on the positive (A1, A4, A6).
5.Dealing with death	The need for peace at the end of life	ask about the specific experience of death (A1, A2). comfort relatives and friends (A1). were prepared for the worst (A2).
	The need to die in a preferred way	need to ensure the well-being of their family, to be with significant people and to feel satisfaction with life after death (A2).

#### Theme 1: Being autonomous

This theme was identified in 5 of the 8 included studies. Autonomy refers to an individual’s capacity to make independent decisions and take actions that align with their personal values [38]. Autonomy constitutes a fundamental need for adolescent cancer patients in palliative care. In this context, the autonomous needs of adolescents with cancer manifest in four key dimensions: preserving self-identity against disease-associated labelling, maintaining continuity with pre-illness life patterns, actively participating in medical decision-making processes, and retaining governance over daily living matters.

##### The need to be treated as equals

Adolescents with cancer wanted to be treated as equals and to be recognised as complete individuals, rather than as cancer patients. Participants noted that changes in appearance due to treatment subjected them to significant social pressure [29]. Many longed for “normalised” social interactions; they wished to joke and make videos with friends as before, but felt uncomfortable when changes in their appearance attracted undue attention [30, 35].

##### The need to continue life as usual

Adolescents with cancer harboured a strong desire to maintain a normal life, but physical limitations and changes in appearance due to treatment often excluded them from everyday activities, leading to feelings of isolation. Faced with this challenge, they adopted two typical coping strategies: some participants grappled with self-identity conflicts, rejecting the label “sick” by refusing to be photographed or resisting assistive devices such as wheelchairs [30]. One 13-year-old female explained what made her unhappy was “All my illness: When I see myself in the mirror; when I can’t walk. When I am very dependent” [35]. Other participants sought to reconnect with the world through engaging in creative activities like play and art [30].

##### The need to be informed about their medical condition

Adolescents with cancer showed a need for access to medical information. They were keen to receive detailed information about their diagnosis, the severity of their condition, and the treatment’s prognosis, and they wanted to be kept abreast of their disease’s progression [29, 31]. When healthcare professionals and family members communicated openly using age-appropriate methods, it greatly alleviated the adolescents’ anxiety by giving them a clear understanding of their condition. One participant reported, “My mother was the one who helped me find answers by asking the doctors. Time also helped. My doubts were: how long will it stay like this, how would my recovery be, what would be the type of treatment, various things. Over time, the professionals responded and I became more relaxed, because I learned about things” [31]. Distinct approaches to autonomous decision making have been observed in Western and Asian healthcare contexts. Whereas Western approaches often emphasize patient autonomy, some Asian patients reported limited engagement in decision-making, as the family members and healthcare professionals tended to emphasize that the disease could be cured [29].

##### The need to be in control

Adolescents with cancer expressed a strong desire for autonomy during their treatment; they wished to choose their dietary preferences and have a say in the design of their ward environment. “Some hospital rooms do not have windows, too bad, we have to be separated from the rest, children and old men and women are in a room, a patient under critical conditions should be separated, adolescents should be separated from the rest” [32]. “We should have food diversity; old peoples’ food should not be served for adolescents”. These participants also highlighted the importance of the inpatient environment to their treatment and overall health, expressing a need for healthcare spaces that cater to their age and developmental needs. However, there were individual differences regarding these environmental preferences: some desired a quiet therapeutic space, while others wished for the hospital to provide music, social interactions, or recreational activities [31, 32].

#### Theme 2: Being connected

This theme was consistently identified across all included studies. Connectedness, encompassing the desire of adolescents with cancer to maintain contact with parents, friends, healthcare professionals, and even transcendental elements, is a fundamental need during the palliative care phase. The significance of interpersonal relationships, emotional support, and communication has been extensively explored in the literature. It is evident that connectedness is a universal and crucial behaviour, transcending cultural boundaries.

##### The need to maintain relationships

The social support network of adolescents with cancer—including family, friends, healthcare professionals, and pets—plays a vital role in helping them cope with the challenges of their condition. Adolescents reported that remaining close to these sources of support provides them with joy and relaxation [30, 32, 33]. Additionally, bonding with their pets was particularly valued for the unconditional love and care it offered [30]. Some participants described how treatment disrupted their social lives, leading to a sense of alienation—an involuntary distancing from peers due to hospital isolation and missed shared experiences [29]. This alienation not only hindered social connection but also exacerbated spiritual distress. For example, “They are all in school and I am the only one staying in the hospital. We have little in common.” [29].

##### The need to love and be loved

Adolescence is a period marked by significant social and psychological changes, with individuals often focusing on independence and self-identity. However, for adolescents with cancer, the impact of the disease frequently renders them powerless, increasing their reliance on parents, friends, and healthcare professionals [29, 31, 33]. Some studies reported that support from these groups provided them with courage, strength, and a sense of well-being [30, 31, 35]. However, they were also aware of the stress and pain their condition inflicted on their parents. Consequently, they often tried to present themselves as strong and optimistic, attempting to comfort their parents to alleviate their psychological burden [30, 33, 35]. “I feel sad for my parents who always care for me. They try so hard to stay beside me (F) all this time” [33]. “I’m good, listen to my mother, put away my toys” [30]. “I try to protect my family especially with the gang stuff—family is very, very important to me“ [35].

##### The need to communicate with others

Adolescents with cancer expressed a strong desire to communicate with others, particularly their parents, when confronted with a life-threatening illness, as this provided psychological relief [28–30]. They also sought support from friends, healthcare professionals, and spiritual advisors. Engaging in conversations with these individuals not only empowered them but also encouraged them to reconsider the meaning of life and deepen their understanding of faith [28, 29]. “I’m not a believer, you know, but talking to you makes me feel better” [28]. “My mom told me that I must tell her when I am feeling unhappy. I did. I felt much happier after saying it” [29]. However, some adolescents admitted that despite their need to communicate, they were hesitant to do so for fear of adding to their parents’ burden [29, 32].

##### The need to connect with transcendence

After a cancer diagnosis, some adolescents often connected with transcendence (e.g. God, Buddha, or a force of nature) through prayer, in order to express their fears and seek protection [29, 31, 32]. “I chant the scriptures with my mom every morning and night. The blessing from Buddha makes me feel relieved. The Buddha will be with me and protect me. I don’t feel scared when I chant” [29]. “Prayer helps me, because prayer is nothing more than talking to God, it brings me comfort” [31]. They described prayer as bringing the experience of being close to God and wanted the hospital to provide places for religious activities [32]. Certain participants saw illness as part of God’s plan for their lives and believed that God would eventually heal them [32, 33]. Alternatively, some participants built up spiritual strength against the disease by identifying with the courage of fictional characters in comics and games [29]. However, if they did not get better in the long term, they would begin to reflect on the certainty of their beliefs and start thinking about the possibility of an afterlife [33]. “I was an atheist, but now I prefer to believe in an afterlife”. “I tried [to pray]; it didn’t work. Therefore, I stopped believing in God” [33]. “I am mad at God” [34].

#### Theme 3: Finding the meaning of life

This theme was identified in 4 of the 8 included studies. Adolescents with cancer expressed their search for meaning in the context of their illness by asking existential questions to make sense of the pain experienced and the changes brought about by the illness, as well as a desire to find meaning and fulfilment in the present moment.

##### The need to accept cancer and seek its meaning

Upon receiving a cancer diagnosis, adolescents expressed a profound need to comprehend their illness and life experiences. They often ask existential questions like “Why me?” [28, 29, 31, 33]. Their interpretations of their illness vary; some viewed it through a religious lens, believing it to be “part of God’s plan” while others attributed their condition to karmic retribution for past misdeeds. For example, “I have bullied other children before. That’s why now I am punished” [29]. Others offered more pragmatic explanations, such as dietary factors: “I was a picky eater. I didn’t like to have meat, and so I didn’t have adequate protein. This weakened my immunity system. That’s why I got sick” [29]. In their quest to understand the origins of their cancer, adolescents also reflected on how they perceived their journey with the disease. While some viewed it as a devastating blow that had shattered their family life [33], others saw it as a challenge to be overcome, believing that they will emerge stronger after surviving the disease [29].

##### The need to have a meaningful life

Despite their illness imposing limitations and increasing their dependency on others, adolescents with cancer remained eager to engage in meaningful activities to affirm their self-worth. They found purpose by contributing in any way they can, such as assisting fellow patients or participating in charitable events. For instance, one adolescent shared, “There was a boy in the ward who did not want to talk or eat. So, I talked to him and played games with him. Later, he became happier and more willing to talk. We also ate together. I felt very happy. [Interviewer: Why?] Because I found that I was not useless. It [my life] is still meaningful " [29]. Another expressed a desire to contribute more broadly, saying, “I would like to participate in a charity event” [33].

#### Theme 4: Having a positive attitude

This theme was identified in 5 of the 8 included studies. Adolescents with cancer reported that alongside the physical pain associated with their condition, they were frequently overwhelmed by emotions such as fear, loneliness, and frustration, which generated a strong desire for inner peace. Many state that they value a positive outlook through hope and peace of mind [39–42].

##### The need for peace

Although innovations in medical technology and treatment have improved survival rates among adolescents with cancer, most participants did not experience a sense of inner peace with doctors’ increasing abilities to treat and cure these cancers [3]. In contrast, persistent fear of disease recurrence, treatment side effects, and future uncertainty kept them in a state of continual anxiety, rendering them unable to cease worrying about their illness [43]. For example, “Even when I was extremely sick, I couldn’t stop thinking about the disease. Sometimes, I preferred to jump out of the window to stop everything instead of being tortured " [29]. “I like quiet surrounding, I love a quiet place, I hate crowd. Crowd bother me” [32].

##### The need to sustain hope

Adolescents with cancer have stated that hope not only strengthens them but also sustains them throughout their treatment [28–30]. Hope is commonly viewed as a psychological asset comprising three core dimensions: (1) a biomedical perspective, where the hope for a cure is paramount. For example, “I pay attention to any change in my white blood cell count. When I know the count is stable, it seems that the disease is under control. Then, I feel there is a hope in my life again” [29]. (2) The social dimension, reflecting the aspiration to resume normal life and sustain relationships. The majority of participants reported their hope to continue life as usual [30, 35], maintaining contact with parents, friends, healthcare providers, and transcendental elements [29, 30, 32].(3) The developmental aspect, focusing on achieving future life goals. “When I look ahead, I see another life, only better. After treatment I will be free” [31]. Some participants fostered hope by employing positive emotional regulation strategies or by concentrating on the positives in their lives. For example, “say thank you (to God) every day for at least three pretty things happening, even when the day was terrible, and choose (maximum) three things, that made that day horrible, that I would hope to be able to improve the following day” [28]. “Everything makes me either feel good or the negative is so insignificant that I don’t notice it” [30]. “I felt sick after it (chemotherapy) yet I also felt fortunate, because I was known by many people, I feel very proud” [33].

#### Theme 5: Dealing with death

This theme was identified in 2 of the 8 included studies. Adolescents with cancer expressed the need to confront their fears of death, seeking to understand what happens post-mortem and whether it involves pain. They continually explore the meaning of death and express a desire to be surrounded by loved ones in their final moments.

##### The need for peace at the end of life

During their long-term treatment, adolescents with cancer frequently voiced a profound fear of death and persistently questioned what the actual experience of dying would be like [28, 29]. For example, “What do you think will happen to me after I die? What happens when your eyes close for the last time?” [28]. “Will I feel pain when I die?” [29]. As the illness progresses, some adolescents experienced a shift in their attitudes towards death. They discussed death with their families, began to appreciate the significance of this life event, and started to come to terms with the most daunting outcomes. They also strove to reduce the suffering of those around them [29]. One reflection was, “Even if you haven’t been successful, you’re the best doctors in the world. I know you’ve done everything possible. I’m fine, doctor, seriously. Don’t look so sad. I will die with a smile, remembering the Pope’s smile” [28]. This journey often mirrors a child’s evolving struggle through “fear, exploration, and acceptance,” while also underscoring their yearning for peace at life’s end.

##### The need to die in a preferred way

Terminally ill adolescents frequently articulate distinct psychosocial needs when faced with mortality. These include (1) a profound desire for meaningful presence of loved ones during their remaining time, (2) the pursuit of life closure and satisfaction, and (3) altruistic concerns regarding their family’s future well-being, encompassing both emotional stability and financial security. One adolescent stated, “If I had a few days to live, I would prefer to go to my home, be in the comfort of my family, the people I love, to attend church. Because there I know that there are people who really love me, who know me, who have a real affection for me and I would prefer to die with people like that” [31]. “When someone dies, the professional must give support to the family by talking” [31].

## Discussion

This review explores, compares, and synthesizes eight qualitative studies on the spiritual needs of adolescents with cancer, identifying various themes.

### Being autonomous

The autonomous needs of adolescents with cancer reflect their efforts to resolve the identity conflict between being a “passive patient” and an “independent individual.” The painful treatments, hospitalisation, and isolation associated with cancer can lead young people to feel that their lives are completely out of their control. They seek to regain a sense of psychological control through making small choices—such as about medical information, dietary preferences, and environmental settings—to alleviate feelings of helplessness and emotional distress [44].

Previous research demonstrates that adolescents with cancer who acquire autonomy in disease management, encompassing both a comprehensive understanding of their health status and active engagement in clinical decision-making, experience improved transitions to adult healthcare services [45, 46]. In Asian cultures, autonomous decision-making is typically expressed through family consensus, with the Confucian principle of “filial piety” framing medical choices as collective family responsibilities. Healthcare professionals in these regions often adopt protective disclosure strategies that prioritize hope preservation over full prognostic transparency [47]. Therefore, family members, friends, and healthcare professionals should avoid solely defining them as patients and instead respect their individuality. Healthcare professionals can offer limited choices, such as deciding the order of tests, to enhance their psychological state and facilitate overall recovery.

### Being connected

Connectedness includes emotional ties to others, such as family, friends, and healthcare professionals, as well as a connection to transcendental elements like God, Buddha, or natural forces. For adolescents with cancer, the inherent need for connectedness is crucial for their psychological development and serves as a vital coping mechanism in dealing with the trauma of their illness [48, 49]. Cancer treatment often forces these adolescents into social isolation, marked by halted academic progress and drastically reduced interactions with peers, which can lead to profound feelings of loneliness [50]. Maintaining connections with family and friends not only helps alleviate their emotional distress but also bolsters treatment adherence through the co-creation of meaning [51]. In addition, some participants indicated that pets could alleviate their emotional distress. Similar benefits have been demonstrated by other groups, such as Berry et al. [52], who noted that pet therapy significantly reduced stress and disruptive behaviour in children with autism spectrum disorders. Additionally, adolescents with cancer often feel a compelling need to “help others,” reflecting a psychosocial adjustment strategy to manage their disease-related trauma. Through acts of helping, they forge a dual identity as both “survivor” and “helper,” which helps counter the negative impacts of stigma and fosters the development of positive social identities and new meaning in their lives [53].

The spiritual world of adolescents with cancer often undergoes significant changes in response to the disease. Some may actively seek a connection to transcendence (e.g. God, Buddha, or a force of nature) as a vital means of making sense of their suffering and regaining a sense of control [34]. Their understanding of, and need for, transcendence is deeply influenced by their cultural backgrounds. Different religions, belief systems, and cultural traditions shape their perceptions of illness, suffering, and divine power, which in turn influences their spiritual practices. Chinese adolescents, for example, often seek protection from their ancestors and the Chinese zodiac, likely because many in China are non-religious and engage in traditional practices passed down through generations [54]. In Lithuania, children’s spiritual expressions tend to prioritize personal meditation and animal companionship rather than participation in organized religion [30]. In contrast, the six studies conducted in countries with deeply religious cultures—such as Italy, Brazil, Indonesia, and the United States—show that affected adolescents are more likely to turn to their own belief systems (e.g. Christianity, Islam) to find spiritual refuge and strength to cope with their illness. Previous studies have shown that prayer is a central religious practice for children with cancer across many cultures. However, most healthcare facilities lack dedicated spaces for religious practices and support materials [32]. This highlights the need for clinical healthcare professionals to respect the child’s religious beliefs and cultural background and to provide access to natural or religious resources. For example, enabling the adolescents and their family to partake in religious activities consistent with their beliefs and inviting religious figures, approved by the healthcare facility, to offer spiritual comfort to the adolescent and family.

### Finding the meaning of life

Adolescents expressed their search for meaning within the context of their illness, providing various interpretations for the causes of cancer, which often reflect cultural influences. Adolescents with religious beliefs typically associate their illness with the will of the gods, seeing it as a life plan or a test. Those influenced by traditional Chinese folk beliefs might explain their illness in terms of concepts such as karma - a central tenet in Buddhism referring to the cycle of cause and effect [55]. Serious diseases like cancer are often seen as punishment for misdeeds committed in this or previous lives, or as collective moral failures of ancestors. Consequently, unlike Western adolescents who may feel anger towards God, some Chinese adolescents and their families might experience shame about having the disease. Apart from seeking the reasons for cancer, participants expressed diverse interpretations of their cancer experiences. While some perceived the illness as a profoundly disruptive life event, others viewed it as a catalyst for post-traumatic growth, wherein adversity fostered psychological development. A salient theme emerged among some Chinese adolescent participants: the perception of cancer as a familial financial burden—with some even wanting to give up their treatment. Traditional filial piety values could account for this perception. This concept emphasises children’s obedience and self-sacrifice to earn parental approval while avoiding bringing hardship [5, 6]. Paediatric healthcare professionals should assess the cultural attributions and emotional states of adolescents with cancer, focusing particularly on those who experience feelings of self-blame or shame, and provide culturally appropriate interventions. For those with religious or karmic attributions, professionals could engage the hospital’s spiritual care team or relevant religious figures to help reframe the illness as a “growth test” rather than a “moral punishment.” For those troubled by financial impacts, support could integrate Confucian ethics - emphasizing treatment adherence as authentic filial piety expression, alongside practical medical aid assistance to alleviate family strain and promote post-traumatic growth.

### Having a positive attitude

Adolescents with cancer often experience prolonged distress from anxiety, loneliness, and the uncertainty of treatment, which intensifies their need for peace and hope. Hockley posits that hope constitutes not merely future-oriented expectation, but rather an active process integrating conscious goals with unconscious meaning-making [56]. Chochinov, et al. reported that hope was related to concepts of meaning and purpose. For patients nearing death, maintaining hope was intimately connected with a sense that life continued to serve some purpose or held meaning enough to sustain their continued existence [57]. Mack et al. [58] found that levels of hope were significantly and positively correlated with levels of psychological peace. Increased levels of hope can notably reduce anxiety and depression, and improve mental health [59]. Consequently, taking into account individual differences, paediatric healthcare professionals can alleviate the constraints of the “patient identity” through emotional de-escalation and narrative therapy. This approach helps maintain psychological balance during treatment and cultivates a sustainable sense of hope.

### Dealing with death

Adolescents with cancer often express a need to manage their fear of death and their wish to spend their final days with their families, in a manner of their choosing [31]. This fear of death may stem from a limited understanding of what death entails. Their questions about death, such as “Will I feel pain when I die?“, often arise from trying to comprehend this abstract concept. Most participants expressed a strong desire to end their lives with the autonomy to choose their surroundings and to be with their families. Therefore, when responding to an adolescent’s questions about death, it is crucial to provide honest yet age-appropriate answers, taking care not to over emphasise the details. It is also important to consider each adolescent’s unique experiences, cultural background, and spiritual needs and to refer them for professional psychological support if necessary.

### Advantages and limitations

Currently, the majority of research has concentrated on the spiritual needs, spiritual health, and spiritual care of adults or older adults, with minimal focus on adolescents with cancer. This review specifically addresses the spiritual needs of adolescents with cancer and offers valuable insights using a meta-synthesis approach that systematically integrates themes from relevant qualitative studies across different regions. However, this review has several limitations. First, interpretive power is limited by the analytical depth of the primary study. Participants in the eight included studies were not systematically grouped by developmental stage (e.g. children aged 6–12 years and adolescents aged 10–19 years), and they covered a broad age range (5–20 years). Given that different age groups exhibit distinct understandings and expressions of spirituality, the current synthesis may not fully capture the nuanced needs of specific developmental stages. In addition, the included studies (*n* = 8) showed heterogeneity in reporting cultural context, with some providing only the study country. This limited our ability to analyze cultural factors in the meta-synthesis. Second, this study aimed to explore the spiritual needs of adolescents with cancer and found that age and religious beliefs may influence their needs. To date, research on spiritual needs among this population in the Asian cultural context remains scarce. Third, only qualitative studies published in English and Chinese were included, and grey literature was not considered, which may introduce selection bias.

### Future research

Most studies included in this review have focused exclusively on adolescents with cancer, without specifying the type of cancer or the stage of disease progression. Although these young people face common challenges, variations in cancer type, severity, and treatment can lead to differing spiritual needs. Future research should investigate the spiritual needs of adolescents at various stages of cancer development to better understand how these needs evolve over time. Additionally, spirituality is a multidimensional concept that is significantly shaped by cultural context and religious beliefs. There may be considerable differences in the understanding and needs related to spirituality among different cultural groups. Researchers should consider developing culturally sensitive spiritual assessment tools based on our findings.

## Conclusion

Spirituality plays a crucial role in the lives of adolescents with cancer, significantly impacting their spiritual health and quality of life. Yet, the spiritual needs of this demographic are frequently neglected. There is an urgent need for a multidisciplinary approach that includes psychology, nursing, and clinical medicine to provide a framework for paediatric healthcare professionals to develop targeted interventions that address the spiritual needs of adolescents with cancer. This study indicates that adolescents with cancer’s spiritual needs vary across cultural backgrounds and differ from those of adult patients. Therefore, the formulation of a spiritual care plan should consider the adolescent’s age, developmental stage, spiritual and religious beliefs, and socio-cultural context.

## Supplementary Information

Supplementary Material 1.

Supplementary Material 2.

## Data Availability

All data generated or analysed in this study are included in the published article [and its supplementary information files].

## Abbreviations

PRISMA: Preferred Reporting Items for Systematic Reviews and Meta-synthesis
