# Supplementary material for: Spiritual needs of adolescents with cancer: a systematic review and meta-synthesis
Source: BMC Palliat Care. 2025 Oct 15;24:257. doi: 10.1186/s12904-025-01904-1 (PMC12523037; doi:10.1186/s12904-025-01904-1)
Supplement: Supplementary file 1 — Supplementary Material 1. [file 12904_2025_1904_MOESM1_ESM.docx]

Table S1. Search Strategy

| **Databases** | **Search  No.** | **Query** | **Results** |
| --- | --- | --- | --- |
| **PubMed (MeSH)** | #1 | Search: "Neoplasms"[Majr] Sort by: Most Recent | 3,628,64 |
|  | #2 | Search: 'Benign Neoplasm'[Title/Abstract] OR 'Benign Neoplasms'[Title/Abstract] OR Cancer[Title/Abstract] OR Malignancy[Title/Abstract] OR Malignant Neoplasm[Title/Abstract] OR Malignant Neoplasms[Title/Abstract] OR Neoplasia[Title/Abstract] OR Neoplasm[Title/Abstract] OR 'Neoplasms, Benign'[Title/Abstract] OR Tumor[Title/Abstract] OR Tumors[Title/Abstract] | 3,639,657 |
|  | #3 | Search: ("Neoplasms"[Majr]) OR ('Benign Neoplasm'[Title/Abstract] OR 'Benign Neoplasms'[Title/Abstract] OR Cancer[Title/Abstract] OR Malignancy[Title/Abstract] OR Malignant Neoplasm[Title/Abstract] OR Malignant Neoplasms[Title/Abstract] OR Neoplasia[Title/Abstract] OR Neoplasm[Title/Abstract] OR 'Neoplasms, Benign'[Title/Abstract] OR Tumor[Title/Abstract] OR Tumors[Title/Abstract]) | 4,933,387 |
|  | #4 | Search: "Adolescent"[Majr] Sort by: Most Recent | 5,556 |
|  | #5 | Search: Adolescence[Title/Abstract] OR Adolescents[Title/Abstract] OR 'Adolescents, Female'[Title/Abstract] OR 'Adolescents, Male'[Title/Abstract] OR Teenagers[Title/Abstract] OR Teens[Title/Abstract] OR Youth[Title/Abstract] | 411.146 |
|  | #6 | Search: ("Adolescent" [Mar]) OR (Adolescence[Title/Abstract] OR Adolescents[Title/Abstract] OR 'Adolescents, Female'[Title/Abstract] OR 'Adolescents, Male'(Title/Abstract] OR Teenagers[Title/Abstract] OR Teens[Title/Abstract] OR Youth[Title/Abstract]) | 413,177 |
|  | #7 | Search: "Child" Majr] Sort by: Most Recent | 3,978 |
|  | #8 | Search: Children[Title/Abstract] OR minors[Title/Abstract] | 1,366,369 |
|  | #9 | Search: ("Child"[Majr]) OR (Children[Title/Abstract] OR minors[Title/Abstract]) | 1,368,020 |
|  | #10 | Search: (("Adolescent"[Majr]) OR (Adolescence[Title/Abstract] OR Adolescents[Title/Abstract] OR 'Adolescents, Female'[Title/Abstract] OR 'Adolescents, Male'[Title/Abstract] OR Teenagers [Title/Abstract] OR Teens[Title/Abstract] OR Youth[Title/Abstract])) OR (("Child"[Majr]) OR (Children[Title/Abstract] OR minors[Title/Abstract])) | 1,624,969 |
|  | #11 | Search: spirituality[Title/Abstract] OR ‘spiritual needs’[Title/Abstract] OR ‘spiritual need’[Title/Abstract] OR "religious needs"[Title/Abstract] OR "religious need"[Title/Abstract] OR religion[Title/Abstract] OR Sensitivity, Spiritual[Title/Abstract] OR ‘Spiritual Sensitivity’ [Title/Abstract] OR Spiritualities[Title/Abstract] | 11,359 |
|  | #12 | Search:'Grounded Theory'[Title/Abstract] OR 'Phenomenological Research'[Title/Abstract] OR  Interview [Title/Abstract] OR 'Semi-structured Interview'[Title/Abstract] OR 'In-depth Interview'[Title/Abstract] OR 'Focus Group Discussion'[Title/Abstract] OR 'Observation Research'[Title/Abstract] OR qualitative[Title/Abstract] | 540,898 |
|  | #13 | Search: Search: (((("Neoplasms"[Majr]) OR ('Benign Neoplasm'[Title/Abstract] OR 'Benign Neoplasms'[Title/Abstract] OR Cancer[Title/Abstract] OR Malignancy[Title/Abstract] OR Malignant Neoplasm[Title/Abstract] OR Malignant Neoplasms[Title/Abstract] OR Neoplasia[Title/Abstract] OR Neoplasm[Title/Abstract] OR 'Neoplasms, Benign'[Title/Abstract] OR Tumor[Title/Abstract] OR Tumors[Title/Abstract])) AND ((("Adolescent"[Majr]) OR (Adolescence[Title/Abstract] OR Adolescents[Title/Abstract] OR 'Adolescents, Female'[Title/Abstract] OR 'Adolescents, Male'[Title/Abstract] OR Teenagers[Title/Abstract] OR Teens[Title/Abstract] OR Youth[Title/Abstract])) OR (("Child"[Majr]) OR (Children[Title/Abstract] OR minors[Title/Abstract])))) AND (spirituality[Title/Abstract] OR ‘spiritual needs’[Title/Abstract] OR ‘spiritual need’[Title/Abstract] OR "religious needs"[Title/Abstract] OR "religious need"[Title/Abstract] OR religion[Title/Abstract] OR Sensitivity, Spiritual[Title/Abstract] OR ‘Spiritual Sensitivity’[Title/Abstract] OR Spiritualities[Title/Abstract])) AND ('Grounded Theory'[Title/Abstract] OR 'Phenomenological Research'[Title/Abstract] OR Interview [Title/Abstract] OR 'Semi-structured Interview'[Title/Abstract] OR 'In-depth Interview'[Title/Abstract] OR 'Focus Group Discussion'[Title/Abstract] OR 'Observation Research'[Title/Abstract] OR qualitative[Title/Abstract]) | 60 |
| **Cochrane Library** | #1 | MeSH descriptor: Neoplasms explode all trees | 126239 |
|  | #2 | (Neoplasm, Benign OR BnIn Nlasm OR Beni OR Nesms, Benin luor OR lurs OR Neoplasia OR Cancers OR Malignancy OR Cancer OR Neoplasm, Malignant OR Malignant Neoplasms OR Malignancies OR Neoplasms, Malignant OR Malianant Neoplasm):ti.ab.kw | 279907 |
|  | #3 | #1 OR #2 | 293074 |
|  | #4 | MeSH descriptor: Adolescent] explode all trees | 135760 |
|  | #5 | (Adolescents OR Male Adolescents OR Male Adolescent OR Adolescents, Male OR Adolescent, Male OR Adolescence OR Teen OR Teenager OR Teenagers OR Female Adolescents OR Adolescent, Female OR Female Adolescent OR Adolescents, Female OR Youth OR Youths OR child OR children OR minors):ti,ab,kw | 332995 |
|  | #6 | #4 OR #5 | 332995 |
|  | #7 | (spirituality OR spiritual needs OR spiritual care OR spiritual need OR Spiritualism OR Sensitivities, Spiritual OR Spiritual Sensitivities OR Sensitivity, Spiritual OR Spiritual Sensitivity OR Spiritualities OR religious needs):ti,ab,kw | 2650 |
|  | #8 | #3 and #6 and #7 | 86 |
| **Web of  Science** | #1 | TI= (Neoplasms OR "Benign Neoplasm" OR "Benign Neoplasms" OR Cancer OR Malignancy OR "Malignant Neoplasm" OR "Malignant Neoplasms" OR Neoplasia OR Neoplasm OR Neoplasms, Benign OR Tumor OR Tumors) and Preprint Citation Index (Exclude - Database) | 2,982,500 |
|  | #2 | TI=(Adolescence OR Adolescents OR Adolescents, Female OR Adolescents, Male OR Teenagers OR Teens OR Youth OR child OR children OR minors) and Preprint Citation Index (Exclude - Database) | 1,642,723 |
|  | #3 | TI= ("spiritual need" OR "spiritual needs" OR "religious needs" OR "religious need" OR spirituality OR religion) and Preprint Citation Index (Exclude -Database) | 31,311 |
|  | #4 | TS= (Grounded Theory OR Phenomenological Research OR Interview OR Semi-structured Interview OR In-depth Interview OR Focus Group Discussion OR Observation Research OR qualitative and Preprint Citation Index (Exclude -Database) | 2,366,118 |
|  | #5 | #1 AND #2 AND #3 AND #4 and Preprint Citation Index | 18 |
| **MEDLINE** | #1 | TI=(Neoplasms OR "Benign Neoplasm" OR "Benign Neoplasms" OR Cancer OR Malignancy OR "Malignant Neoplasm" OR "Malignant Neoplasms" OR Neoplasia OR Neoplasm OR Neoplasms, Benign OR Tumor OR Tumors) | 2,027,005 |
|  | #2 | TI=(Adolescence OR Adolescents OR Adolescents, Female OR Adolescents, Male OR Teenagers OR Teens OR Youth OR child OR children OR minors) | 1,025,328 |
|  | #3 | TI=("spiritual need" OR "spiritual needs" OR "religious needs" OR "religious need"OR spirituality OR religion) | 7,275 |
|  | #4 | TS=(Grounded Theory OR Phenomenological Research OR Interview OR Semi-structured Interview OR In-depth Interview OR Focus Group Discussion OR Observation Research OR qualitative) | 857,405 |
|  | #5 | #1 AND #2 AND #3 AND #4 | 11 |
| **CINAHL** | S1 | MH neoplasms | 97,116 |
|  | S2 | TI "Benign Neoplasm" OR "Benign Neoplasms" OR Cancer OR Malignancy OR "Malignant Neoplasm" OR "Malignant Neoplasms" OR Neoplasia OR Neoplasm OR Neoplasms, Benign OR Tumor OR Tumors | 470,515 |
|  | S3 | S1 OR S2 | 508,742 |
|  | S4 | ((MH Adolescent) OR (TI (Adolescents OR Male Adolescents OR Male Adolescent OR Adolescents, Male OR Adolescent, Male OR Adolescence OR Teen OR Teenager OR Teenagers OR Teens OR Female Adolescents OR Adolescent, Female OR Female Adolescent OR Adolescents, Female OR Youth OR Youths OR child OR children)) | 457,991 |
|  | S5 | TI (spirituality OR "spiritual needs" OR "spiritual need" OR "spiritual care" OR religion OR "religious needs" OR "religious need") | 8,176 |
|  | S6 | AB Grounded Theory OR Phenomenological Research OR Interview OR Semi-structured Interview OR In-depth Interview OR Focus Group Discussion OR Observation Research OR qualitative | 324,449 |
|  | S7 | S3 AND S4 AND S5 AND S6 | 8 |
| **EMBASE (emtree)** | #1 | neoplasms'/exp | 6,564,136 |
|  | #2 | benign neoplasm':ti OR 'benign neoplasms':ti OR cancer:ti OR malignancy:ti OR 'malignant neoplasm':ti OR 'malignant neoplasms':ti OR neoplasia:ti OR neoplasm:ti OR 'neoplasms, benign':ti OR tumor:ti OR tumors:ti | 2,546,790 |
|  | #3 | #1 OR #2 | 2,065,423 |
|  | #4 | adolescent'/exp | 1,196,275 |
|  | #5 | adolescence:ti OR adolescents:ti OR 'adolescents, female':ti OR 'adolescents, male':ti OR teenagers:ti OR teens:ti OR youth:ti OR child:ti OR children:ti OR minors:ti | 8079 |
|  | #6 | #4 OR #5 | 607,923 |
|  | #7 | spiritual need':ti OR 'spiritual needs':ti OR spirituality:ti OR 'religious needs':ti OR 'religious need':ti OR religion:ti | 6,796,053 |
|  | #8 | grounded theory':ab OR 'phenomenological research':ab OR interview:ab OR 'semi-structured interview':ab OR 'in-depth interview':ab OR 'focus group discussion':ab OR 'observation research':ab OR qualitative:ab | 2,829,182 |
|  | #9 | #3 AND #6 AND #7 AND #8 | 17 |
| **PsycINFO** | S1 | DE Neoplasms | 50,227 |
|  | S2 | TI (Benign Neoplasm OR Benign Neoplasms OR Cancer OR Malignancy OR Malignant Neoplasm OR Malignant Neoplasms OR Neoplasia OR Neoplasm OR Neoplasms, Benign OR Tumor OR Tumors) | 50,057 |
|  | S3 | S1 OR S2 | 65,975 |
|  | S4 | TI Adolescents OR Male Adolescents OR Male Adolescent OR Adolescents, Male OR Adolescent, Male OR Adolescence OR Teen OR Teenager OR Teenagers OR Teens OR Female Adolescents OR Adolescent, Female OR Female Adolescent OR Adolescents, Female OR Youth OR Youths OR child OR children OR minors | 519,784 |
|  | S5 | TI spirituality OR "spiritual needs" OR "spiritual need" OR "spiritual care" OR religion OR "religious needs" OR "religious need" | 15,157 |
|  | S6 | AB Grounded Theory OR Phenomenological Research OR Interview OR Semi-structured Interview OR In-depth Interview OR Focus Group Discussion OR Observation Research OR qualitative | 492,578 |
|  | S7 | S3 AND S4 AND S5 AND S6 | 7 |
